# Sertraline Treatment Can Mimic Niemann‐Pick Type C Biomarker Profile: A Diagnostic Pitfall

**DOI:** 10.1002/acn3.70411

**Published:** 2026-04-27

**Authors:** Maria Makrygianni, Cecile Pagan, Antony Citterio‐Quentin, Isabelle Rouvet, Giulia Dingeo, Cyril Hanin, Claudine Laurent‐Levinson, Samia Pichard, Bénédicte Héron, Clément Gourguechon, Daniele Mandia, Jerome Guitton, Carole Lacout, Nicolas Schleinitz, Florient Potier, Olivier Flabeau, Elsa Besse‐Pinot, Foudil Lamari, Magali Pettazzoni, Yann Nadjar

**Affiliations:** ^1^ Neuro‐Metabolism Unit, Reference Center for Metabolic and Lysosomal Neurological Diseases, Neurology Department Hôpital Pitié‐Salpêtrière Paris France; ^2^ Parkinson's Disease and Movement Disorders Dept HYGEIA Hospital Athens Greece; ^3^ Biochemistry and Molecular Biology Department Hospices Civils de Lyon Bron France; ^4^ Filière Maladies Héréditaires du Métabolisme (G2M) France; ^5^ Centre de Biotechnologie Cellulaire et biothèque CRB HCL Hospices Civils de Lyon France; ^6^ Department of Biochemistry of Neurometabolic Diseases Pitié‐Salpêrière University Hospital, APHP Paris France; ^7^ Child Psychiatry Department Hôpital Pitié‐Salpêtrière Paris France; ^8^ Laboratory of Childhood Genetic Diseases Sorbonne University, Armand Trousseau Hospital Paris France; ^9^ Inborn Errors of Metabolism Clinical Unit Necker Hospital Paris France; ^10^ Department of Pediatric Neurology, Reference Center for Lysosomal Diseases Armand Trousseau Hospital and FHU I2D2, APHP Sorbonne University Paris France; ^11^ Clinical Hematology Department University Hospital Amiens France; ^12^ Department of Internal Medicine University Hospital Center Angers France; ^13^ Department of Internal Medicine University Hospital Center (CHU) of Marseille – Timone Hospital Marseille France; ^14^ Department of Neuro‐Cognition and Neuro‐Ophthalmology University Hospital Center (CHU) Lyon Lyon France; ^15^ Department of Neurology Hospital Center of Côte Basque Bayonne France; ^16^ Department of Clinical Genetics and Neurology Clermont‐Ferrand University Hospital Clermont‐Ferrand France

**Keywords:** biomarkers, Niemann‐pick type C, sertraline

## Abstract

**Background:**

Oxysterols (cholestane‐3β,5α,6β‐triol and 7‐ketocholesterol) and N‐palmitoyl‐O‐phosphocholineserine (PPCS) are sensitive biomarkers for Niemann‐Pick disease type C (NPC) screening. However, false‐positive results occur, with a biomarker profile suggestive of NPC despite the absence of pathogenic variants in genes involved in NPC or other inborn errors of metabolism.

**Objective:**

To identify causes of false‐positive biomarker profiles mimicking NPC.

**Methods:**

We conducted a multicenter retrospective study of 15 patients with false‐positive oxysterols and PPCS profiles referred between 2017 and 2022 to two French NPC reference laboratories. Clinical data were collected via standardized chart review. The impact of Sertraline on NPC‐like biological features was evaluated using the filipin test in fibroblasts and biomarker analysis in sertraline‐treated patients.

**Results:**

Thirteen of 15 patients with false‐positive biomarkers were treated with sertraline. Two patients who discontinued sertraline showed normalization of biomarkers. The filipin test revealed that Sertraline disrupts intracellular cholesterol trafficking, a hallmark of NPC cellular features. Finally, among 47 sertraline‐treated patients without NPC‐suspicion, 26 (55%) had biomarker profile mimicking NPC.

**Interpretation:**

Sertraline use is frequently associated with elevated biomarkers that mimic NPC, representing a primary cause of false‐positive results in NPC screening. Genetic analysis of *NPC1* and *NPC2* remains essential to confirm NPC diagnosis. Most sertraline‐treated patients with false‐positive biomarkers presented predominantly atypical psychiatric symptoms, though one exhibited a clinical picture highly suggestive of NPC following prolonged sertraline exposure. The long‐term clinical effects of sertraline use need further evaluation.

## 1 Introduction

**FIGURE 1:**
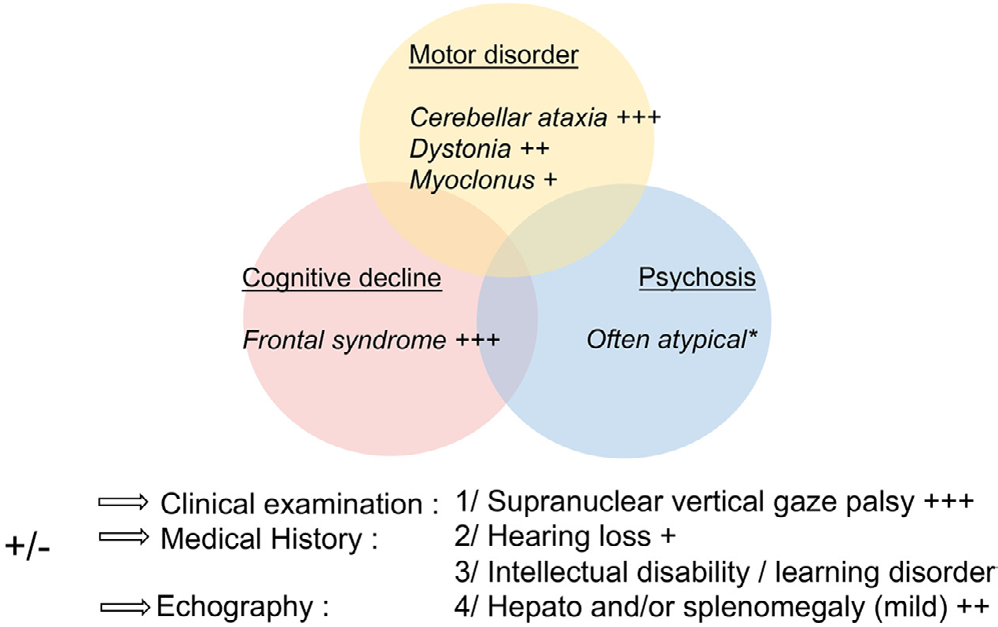
Clinical presentation of adolescent and adult‐onset NP‐C disease. Patients can enter the disease in three different clinical syndromes that initially may be isolated or may overlap: (i) cognitive decline with mainly frontal syndrome; (ii) psychosis (often atypical*, meaning with associated intellectual disability, and/or unusual cognitive impairment, and/or visual hallucinations, and/or other neurological manifestations, and/or resistance to antipsychotic treatment); and (iii) motor disorder (from cerebellar dysfunction ± dystonia ± myoclonus). At presentation, these syndromes may be associated to other signs that can argue for the diagnosis: At clinical examination, vertical supranuclear gaze palsy is highly suggestive of NP‐C disease; history of hearing loss and/or intellectual disability; and finally, echographic finding of hepatomegaly and/or splenomegaly.

Niemann‐Pick disease type C (NPC) is a rare, neurodegenerative, autosomal recessive lysosomal disorder characterized by the accumulation of unesterified cholesterol and other lipids in the late endosome/lysosome, due to a defect of NPC1 (95% of cases) or NPC2 (5% of cases) protein (NPC1 OMIM# 257,220 or NPC2 OMIM# 607,625). The accumulation of lipid leads to a complex disease with multisystem involvement, including neurological, psychiatric, and visceral symptoms. The clinical presentation is highly variable, and its severity correlates with the age at onset of neurological symptoms (see Figure 1 for the clinical presentation of adolescents and adults with NPC) [1]. Accurate and early diagnosis is critical, given the progressive nature of the disease and the importance of early treatment to improve outcomes.

Currently, three plasma biomarkers—cholestane‐3β,5α,6β‐triol (C‐triol), 7‐ketocholesterol (7‐KC), and N‐palmitoyl‐O‐phosphocholine serine (PPCS, belonging to a new class of lipids, formerly called lysosphingomyelin 509) are used as first‐line tests for NPC screening, offering advantages over historical cellular methods, i.e., the filipin staining test, highlighting an accumulation of unesterified cholesterol in cultured fibroblasts, which is invasive, tedious, and less suited for large‐scale screening. These biomarkers exhibit excellent sensitivity and specificity (above 90%), with PPCS demonstrating the highest accuracy in distinguishing NPC from controls and carrier subjects [2]. However, both C‐triol and 7‐KC have been reported to be elevated in different conditions such as acid sphingomyelinase deficiency (ASMD), lysosomal acid lipase deficiency (LAL‐D) and cerebrotendinous xanthomatosis (CTX), whereas 7‐KC alone was found elevated in Smith–Lemli–Opitz syndrome (SLO) [3, 4]. The elevation of these biomarkers, with or without a moderate elevation of lysosphingomyelin, is characteristic of NPC patients, while a more substantial elevation of LysoSM is generally found in acid sphingomyelinase deficiency (ASMD) [4, 5].

In this study, we report 15 patients with increased biomarkers mimicking NPC profile, but for whom genetic testing did not confirm NPC (no pathogenic or likely pathogenic variants identified in *NPC1* or *NPC2*). Other genetic conditions associated with increased biomarkers, including ASMD, LAL‐D, and CTX were also ruled out. We aimed to unveil a possible common cause explaining this biochemical phenotype by retrospectively analyzing clinical and biological data. We show that treatment with sertraline, a widely‐prescribed antidepressant, is mostly responsible for the false‐positive biomarker profile and disrupts intracellular cholesterol trafficking, as in NPC patients.

## 2 Patients And Methods

This was a multicenter, retrospective study conducted in France. All described procedures below were performed as part of standard care. The only exception was the NPC biomarker assays in ‘sertraline‐treated’ patients, which were performed in the residual plasma samples after sertraline measurement (see below).

### 2.1 Patients

*“Pseudo‐NPC” patients* were first identified as having elevated levels of at least two biomarkers (one of C‐triol or 7‐KC, and PPCS), with no pathogenic or likely pathogenic variant identified in *NPC1*, *NPC2*, or *SMPD1* genes. All patients' blood samples were referred between 2017 and 2022 from various French centers to either the metabolic biochemistry laboratory at the Pitié‐Salpêtrière Hospital in Paris (HPSL) or the biochemistry and molecular biology department at the Lyon University Hospital (HCL), the reference laboratories for NPC screening in France. We retrospectively conducted a structured review of the available medical charts to gather demographic, clinical, and pharmacological data, and the results of the laboratory tests performed at the time of biomarker measurement.

“*Sertraline‐treated” patients* refer to a group of 47 patients who were referred to the Biochemistry and Molecular Biology department of Lyon University Hospital between July and September 2024 for sertraline measurement as part of therapeutic monitoring, with no clinical suspicion of NPC, for whom sertraline concentrations were above the therapeutic lower limit value (i.e., > 10 μg/L).

*“Child psychiatry” patients* refer to a group of patients who were hospitalized in the Child Psychiatry Department of Pitié‐Salpêtrière Hospital between 2023 and 2024 and had been screened for NPC biomarkers for clinical reasons. We retrospectively collected clinical and biological data for these patients, including psychiatric diagnoses and prescribed drugs.

### 2.2 NPC Biomarker Assays

C‐triol and 7‐KC (oxysterols), PPCS, and Lyso‐SM were measured in EDTA plasma by liquid chromatography coupled with tandem mass spectrometry in two reference laboratories (HPSL and HCL).

Values were normalized in multiples of cutoff (MoC) to harmonize results between the two laboratories; thus, the reference range is ≤ 1.

We compared the concentrations of plasma biomarkers at the time of the first biochemical sampling in “pseudo‐NPC” patients with those in genetically confirmed French NPC patients studied by Mandia et al. (*n* = 22) [6].

HPSL methods. For oxysterols, a mixture of deuterated C‐triol and 7‐KC in methanol was added to plasma samples, and lipids were extracted with hexane. After evaporation, the extracted oxysterols were derivatized with picolinic acid [7, 8]. After a second extraction with hexane and evaporation, the residue was redissolved in methanol and injected into the UPLC–MSM system (Shimadzu 8060‐NX) for oxysterol quantification in multiple reaction monitoring, positive mode. For PPCS and Lyso‐SM, a mixture of Lyso‐SM18‐d9 and PPCS‐d9 in methanol was mixed with plasma. After mixing and centrifugation, the supernatant was injected into the UPLC–MSM system (Shimadzu 8060‐NX) for PPCS and Lyso‐SM quantification.

HCL methods. Lyso‐SM and PPCS were concomitantly measured in 200 μL of plasma, following exactly (including equipment) a previously described procedure [9]. C‐triol was quantified using a published method with minor modifications [10]. In brief, a deuterium‐labeled internal standard was added to 50 μL of plasma, and dimethylaminobutyrate derivatization was performed after ethyl acetate extraction. Samples were injected onto a LC‐20ad HPLC (Shimadzu) equipped with a PFP column (Agilent) and coupled with an API 4500 QTRAP triple quadrupole (AB Sciex LLC, Framingham, USA) instrument with ESI ionization. Quantification was performed using Analyst software.

### 2.3 Sertraline Measurement

An Acquity UPLC system coupled with a Xevo TQ‐S Micro (Waters, USA) triple quadrupole MS equipped with an electrospray Z‐spray source was used. Sertraline and its internal standard sertraline‐D3 were monitored in positive ion mode and multiple reaction monitoring (MRM) mode with respectively two transitions m/z 306.1 → 159.0 (used for quantification) and m/z 306.1 → 275.1 (used as qualifier ions) for sertraline and one transition m/z 309.2 → 159.0 for sertraline‐D3. Samples were separated on an Acquity UPLC BEH C18 1.7 μm 2.1 × 100 mm (Waters, USA) at 50°C with a flow rate of 600 μL/min. Gradient elution was performed with a mobile phase composed of formate buffer (5 mM) (eluent A) and formic acid (0.1%) acetonitrile (eluent B).

### 2.4 Genetic Studies

*NPC1, NPC2, SMPD1, LIPA*, and *CYP27A1* genes were sequenced using targeted massively parallel sequencing. Briefly, DNA was extracted from whole blood using NucleoSpin Blood L100 columns (Macherey‐Nagel). Coding sequences (exons +/− 25 nucleotides) were sequenced on an Illumina NextSeq 500 sequencer after enrichment by capture (SeqCap EZ Choice, NimbleGen, Roche). Data were analyzed with a bioinformatics pipeline developed at HCL, allowing single nucleotide variants and copy number variations detection.

### 2.5 Filipin Staining Test

The filipin staining test was performed in cultured fibroblasts as previously described [11]. Briefly, as a first step, fibroblasts were cultured for two days in a medium enriched with LDL‐depleted fetal calf serum (LPDS). In the second step, fibroblasts were cultured for one day in a medium enriched with purified human low‐density lipoproteins (LDL). Fibroblasts were then fixed and stained with filipin, a fluorescent reagent that reveals unesterified cholesterol. To determine the effect of sertraline and its main active metabolite, norsertraline, on intracellular cholesterol trafficking, the filipin test was performed with the addition of sertraline and norsertraline to the culture medium during both steps (LPDS medium step and human LDL‐enriched step). Two final concentrations of sertraline were tested in culture media, with or without norsertraline: a concentration within the typical range observed in patients treated with therapeutic doses (sertraline 150 μg/L with or without norsertraline 300 μg/L), and a supratherapeutic concentration (sertraline 500 μg/L with or without norsertraline 1000 μg/L).

### 2.6 Statistics

All statistical analyses were performed using RStudio (version 2014.12.0) and Microsoft Excel (version 2501 for Windows 11). Data were initially processed in Excel for initial organization, cleaning, and descriptive statistics. Further statistical analyses and visualizations were conducted using RStudio or Excel. Comparisons between groups were conducted using the Mann–Whitney *U* test and the chi‐square test. Spearman's rank correlation coefficient (Spearman's ρ) and Fisher's exact test were performed to assess associations between variables. A *p* value < 0.05 was considered statistically significant. All statistical procedures followed appropriate methodological and reporting standards.

### 2.7 Ethics

The retrospective study was conducted in accordance with French legislation and authorized by the National Commission on Informatics and Liberty (CNIL No. 2211991). This study was conducted as a retrospective analysis of previously collected samples and clinical data. As such, no new interventions, treatments, or direct interactions with patients were involved. All data and biological samples used in this study were collected as part of routine clinical care and were anonymized prior to analysis.

## 3 Results

### 3.1 Patients With False‐Positive Plasma NPC Biomarkers Profile Have Sertraline Treatment as a Shared Feature

Among all patients whose blood samples were sent to the HPSL and HCL laboratories for NPC screening between 2017 and 2022, we identified 17 “pseudo‐NPC” patients. They had a biomarker profile similar to NPC, and targeted genetic sequencing that did not confirm a diagnosis of NPC while other known causes of increased biomarkers (ASMD, LAL‐D, CTX) were also excluded. Of these, 8 patients were followed at Pitié‐Salpêtrière Hospital, and 9 in other French centers. Two patients were lost to follow‐up, and ultimately, 15 patients (8 females, 7 males) were included in the study.

**FIGURE 2:**
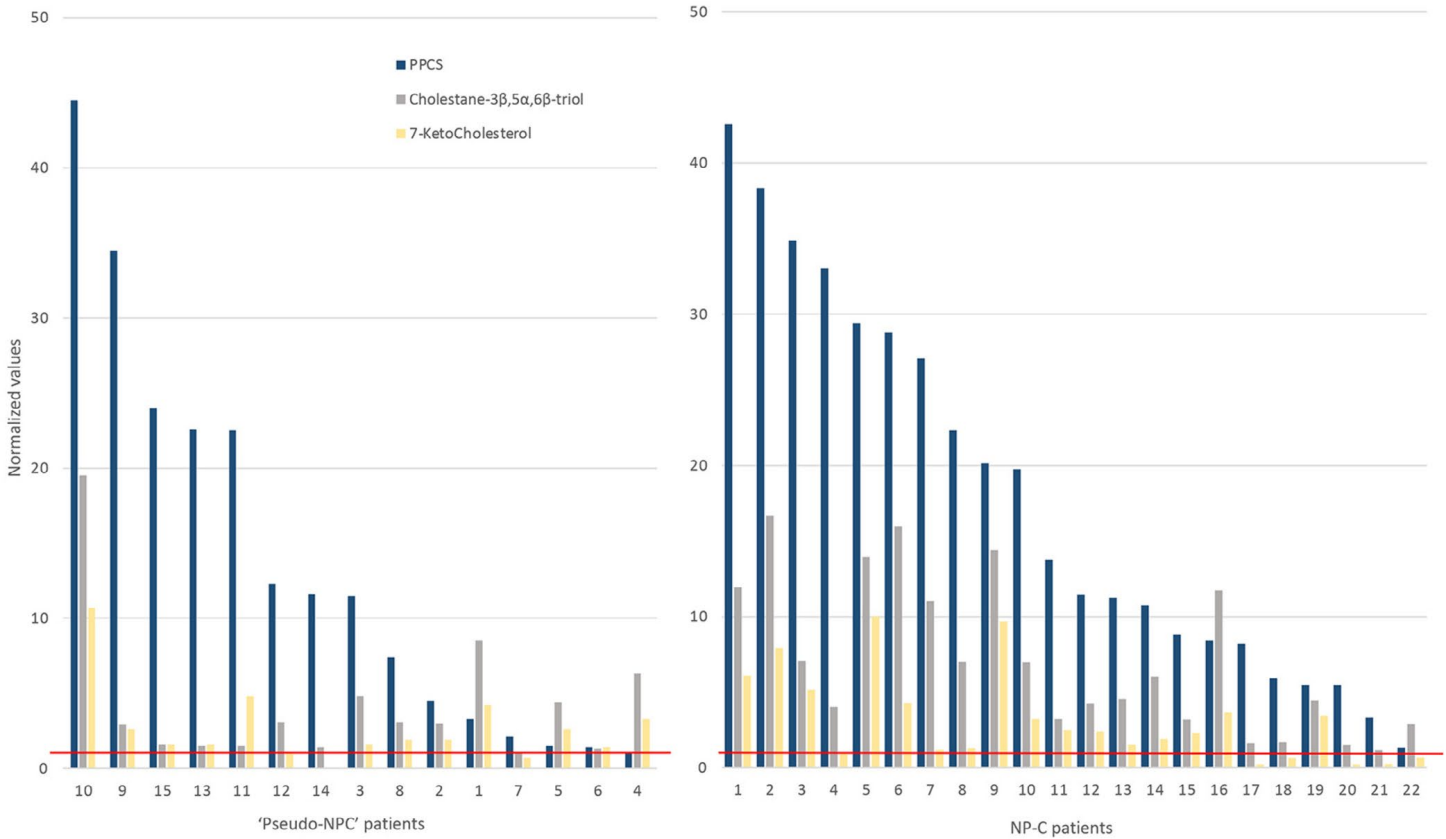
Levels of NPC plasma biomarkers at the first sampling in ‘pseudo‐NPC’ cohort and in a genetically confirmed NPC cohort [6], from highest to lowest N‐palmitoyl‐O‐phosphocholineserine (PPCS) biomarker. The red line represents the normalized upper value for all three biomarkers (i.e., a value above the red line is abnormally high). For patient 14, 7‐KC value was not available.

Multiple samples (> 1) obtained at different time points were available for 8/15 patients, yielding a total of 30 blood samples. Levels of NPC plasma biomarkers at the first sampling in “pseudo‐NPC” cohort (*n* = 15 samples) were similar to confirmed NPC patients' profiles (Figure 2). The normalized biomarker values were as follows: median C‐triol was equal to 3 MoC (min‐max range: 1.1–19.5) for the pseudo‐NPC cohort versus 2.9 MoC (min‐max range: 1.2–16.7) for the NPC cohort (*p* = 0.07); mean 7‐KC was 1.9 MoC (min‐max range: 0.7–10.7) versus 1.7 MoC (0.2–10) respectively (*p* = 0.64); and mean PPCS was 9.2 MoC (1.1–44.5) versus 4.3 MoC (1.3–42.6) respectively (*p* = 0.08).

### 3.2 Clinical Information

The average age of pseudo‐NPC patients at the time of the first sample was 44 years (min‐max: 11–71 years). Three of them were minors at the time of sampling. None had a family history of consanguinity. Three patients had a notable family history in first‐degree relatives, two with psychiatric disorders and one with early cognitive disorder. Clinical data are summarized in Table 1. 11/15 patients presented with atypical psychiatric symptoms as their primary manifestation, associated with cognitive decline (prominent in 2/11 patients, 1 and 15) or intellectual disability, resistance to neuroleptic treatment, or associated with neurological symptoms. Among them, patients 5 and 14 also had a prominent tremor which prompted screening of NPC; and patient 9 had neurological symptoms suggestive of NPC with ataxia, cognitive decline, and especially slowing of vertical oculomotor saccades. 3/15 patients were screened for NPC due to hepatomegaly and/or splenomegaly: patient 10 in a context of unexplained cholestasis; patient 11 also suffered from schizophrenia and was diagnosed as a myeloproliferative syndrome; patient 13 also suffered from ataxia in a context of unexplained peripheral neuropathy. Finally, patient 12 had an unexplained complex neurological disease with cognitive decline and ataxia. Whole‐genome sequencing was performed for four patients and did not allow making a diagnosis. A filipin staining test in fibroblasts was performed for five patients and was normal for all of them.

**TABLE 1:** Clinical manifestations prompting NPC disease screening in “pseudo‐NPC” patients, and results from genome and filipine test when available.

Patients No	Pt 1	Pt 2	Pt 3	Pt 4	Pt 5	Pt 6	Pt 7	Pt 8	Pt 9	Pt 10	Pt 11	Pt 12	Pt 13	Pt 14	Pt 15	Totals	%
Age/sex	67/F	70/F	16/F	15/M	71/M	23/M	11/M	13/F	57/F	53/F	38/F	38/F	62/M	61/M	66/M		
SERTRALINE use	×	×	×	×	×	×	×	×	×	−	×	×	−	×	×	13	87%
Clinically probable NPC	−	−	−	−	−	−	−	−	×	−	−	−	−	−	−	1	6%
**Psychiatric symptoms**																	
Psychosis	×	×	×	×	−	−	−	−	×	−	×	−	−	−	−	6	40%
Depression	×	×	×	×	×	×	×	×	UKN	−	−	×	−	×	×	11	79%
Auditory hallucinations	×	−	−	−	−	−	−	x	−	−	−	−	−	−	−	2	13%
Obsessive compulsive disorder	−	−	×	×	−	−	×	−	−	−	−	−	−	−	−	3	20%
**Atypical symptoms associated with the psychiatric manifestations**																	
Unusual cognitive impairment	×	−	−	−	×	×	×	−	×	UKN	−	−	−	−	×	6	43%
Associated intellectual disability	−	−	−	−	−	−	−	−	×	UKN	−	−	−	−	−	1	7%
Resistance to neuroleptic treatment	×	×	×	×	×	×	−	−	×	UKN	−	−	−	−	−	7	50%
Associated neurological symptoms	×	×	×	×	×	×	−	×	×	UKN	−	×	−	×	×	11	79%
Visual hallucinations	×	×	−	−	−	−	−	×	−	UKN	−	−	−	−	−	3	21%
**Motor impairment**																	
Gait disorder	−	−	−	−	×	−	−	−	×	−	−	×	×	−	−	4	27%
Weakness UL	−	−	−	−	−	−	−	−	−	−	−	×	−	−	−	1	6%
Weakness LL	−	−	−	−	×	−	−	−	−	−	−	×	×	−	−	3	20%
Ataxia	−	−	−	−	×	−	−	−	×	−	−	×	×	−	−	4	27%
Dysmetria	−	−	×	−	−	−	−	−	−	−	−	×	−	−	−	2	13%
Myoclonus	−	−	−	−	−	−	−	−	−	−	−	×	−	−	−	1	6%
Tremor	−	−	−	×	×	−	−	−	−	−	−	−	−	×	−	3	20%
Dystonia	−	−	−	−	−	−	−	−	−	−	−	−	−	−	−	0	0%
Dysarthria	−	−	−	−	−	−	−	−	−	−	−	−	−	−	−	0	0%
Dysphagia	−	−	−	−	−	−	−	−	−	−	−	×	−	−	−	1	6%
Occulomotor disorder	−	−	−	−	−	−	−	−	×	−	−	−	−	−	−	1	6%
Slowing of vertical saccades	−	−	−	−	−	−	−	−	×	−	−	−	−	−	−	1	6%
**Other symptoms present**																	
Deafness	×	−	−	×	−	−	−	−	−	−	−	−	−	−	−	2	13%
Sensitivity deficit	−	−	−	−	−	−	−	−	−	−	−	×	−	−	−	1	6%
Attention deficit	×	×	×	−	×	×	×	×	×	−	−	×	−	−	−	9	69%
Memory deficit	−	×	−	−	×	×	−	−	×	−	−	×	−	−	−	5	31%
Dysexecutif syndrome	×	−	×	−	−	×	−	−	×	−	−	×	−	−	×	6	40%
Dementia	−	−	−	−	−	−	−	−	×	−	−	−	−	−	×	2	13%
Hepatomegaly	−	−	−	×	×	−	−	−	−	×	×	−	−	−	−	4	26%
Splenomegaly	−	−	−	−	−	−	−	−	−	×	×	−	×	−	−	3	20%
**Genetic analysis and filipin test**																	
Genome	**NEG**	ND	ND	**NEG**	ND	ND	ND	**NEG**	ND	**NEG**	ND	ND	ND	ND	ND	4	NA
**Filipin test**	**NEG**	ND	ND	**NEG**	ND	ND	ND	ND	**NEG**	ND	**NEG**	**NEG**	**NEG**	ND	ND	6	NA

Abbreviations: ×, presence of the clinical characteristic; −, absence; NA, not applicable; ND, not done; NEG, negative; UKN, unknown.

### 3.3 Medications

A review of medical records for all “pseudo‐NPC” patients identified 54 molecules in total administered at the time of initial sampling or within the preceding three months. Table 2 includes only those medications taken by at least two patients in the “pseudo‐NPC” cohort. 13/15 patients were receiving antidepressant therapy with sertraline, with doses ranging from 50 to 200 mg per day. 6/15 patients were receiving both sertraline and the antipsychotic drug risperidone.

**TABLE 2:** Drugs prescribed in the pseudo‐NPC cohort, shared by ≥ 2 patients.

	SERTR	FLUO	ARIP	RISP	GABA	MELA	TRI/CHLO	BISO	EPLE	METF	LEVOT
Patient 1	×		×	×							
Patient 2	×			×		×				×	×
Patient 3	×	×		×							
Patient 4	×	×	×	×		×					
Patient 5	×				×					×	
Patient 6	×										
Patient 7	×		×	×		×					
Patient 8	×			×		×					
Patient 9	×										
Patient 10							×	×	×		×
Patient 11	×		×		×		×			×	
Patient 12	×							×	×		
Patient 13											
Patient 14	×										
Patient 15	×										

Abbreviations: ARIP, aripiprazole; BISO, bisoprolol; EPLE, eplerenone; FLUO, fluoxetine; GABA, gabapentin; LEVOT, levothyroxine; MELA, melatonin; METF, metformin; RISP, risperidone; SERTR, sertraline; TRI/CHLO, trihexyphenidyl hydrochloride.

**FIGURE 3:**
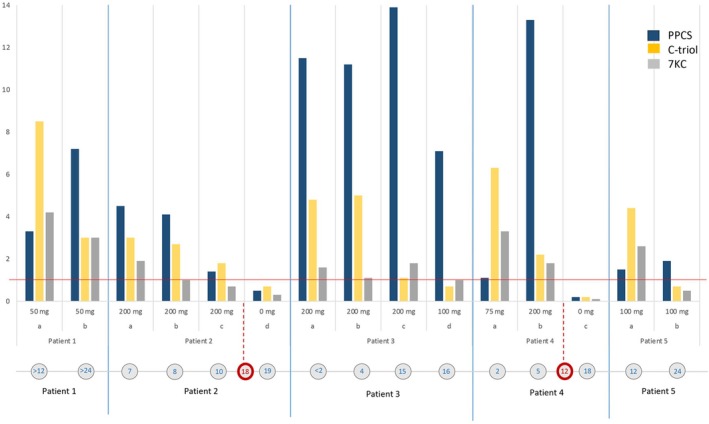
Sertraline dosage in mg at the times of blood sampling for each patient, and biomarker measurements results. a: First sampling, b: Second sampling, c: Third sampling, d: Fourth sampling. Duration of sertraline treatment from the start to time at sampling is indicated in the gray circles in months (> X or < X means the patient had been on sertraline for more, or less respectively, than X months). Dotted red lines represent the timing of sertraline discontinuation, with red circles indicating the delay between start and discontinuation of sertraline. The horizontal red line represents the normalized cut‐off for all three biomarkers.

Sertraline measurement and relationship with biomarkers: Seven out of eight patients for whom multiple samples were collected were on sertraline for at least the first sample. We obtained sufficient data on the duration and the dosage of sertraline for five of them. Changes in biomarker concentrations over time relative to sertraline dosage are illustrated in Figure 3. In patients 2 and 4, biomarker concentrations were normal in the samples collected after sertraline discontinuation.

In order to further explore the hypothesis that sertraline is the cause of false‐positive NPC biomarkers profiles in these 13 pseudo‐NPC patients, we performed three additional studies: (i) we tested NPC biomarkers in plasma from 47 psychiatric patients (“sertraline‐treated” patients) who had no clinical suspicion of NPC but whose sertraline plasma levels were monitored during clinical treatment; (ii) to explore the relevant biological effects of sertraline, we added clinically relevant concentrations of sertraline or its major metabolite in the mediums of cultured fibroblasts from two pseudo‐NPC patients and observed the effect on the filipin staining test that was negative in standard conditions; (iii) in order to consider whether any similar effect might be seen with other commonly used psychotropic drugs, we examined results of NPC biomarker testing in a cohort of 33 “child psychiatry” patients who had been screened for NPC for clinical reasons and who were being treated with a variety of psychotropics.

### 3.4 Patients Treated With Sertraline Display Increased NPC Biomarkers

**FIGURE 4:**
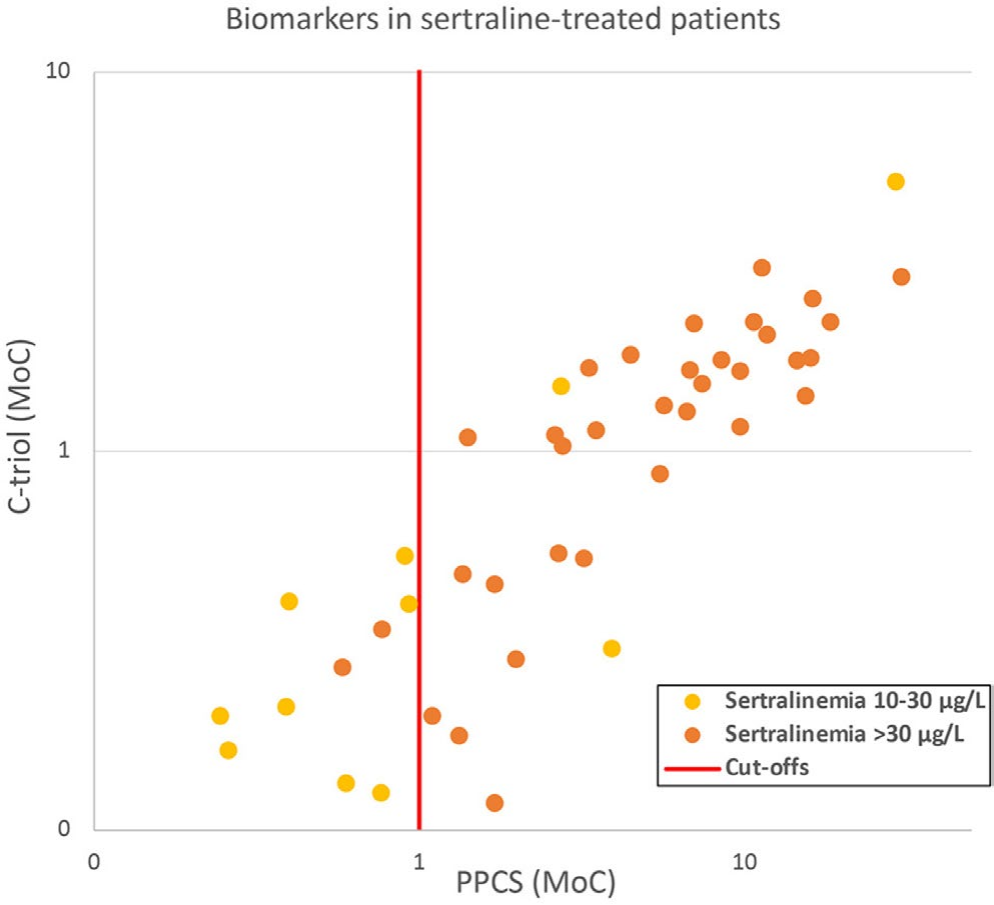
C‐triol and PPCS concentrations from sertraline‐treated patients, according to sertralinemia (bullet color). Red lines represent the upper normal values for C‐triol and PPCS.

Among 47 “sertraline‐treated” patients without clinical suspicion of NPC, C‐triol and PPCS measurement revealed that 26/47 (55%) displayed an NPC‐like profile (with both biomarkers increased), 12/47 (26%) had an atypical profile with only PPCS increased, and only 9/47 (19%) had a normal profile (Figure 4).

In the subgroup with NPC‐like profile, median serum sertraline concentration was 58 μg/L (min‐max: 39–149), while in the subgroup with normal profile median sertraline concentration was 21 μg/L (min‐max: 10–92; *p* < 0.0001, Mann–Whitney *U* test). Considering that therapeutic target for sertralinemia ranges between 10 and 150 μg/L, when serum sertraline concentrations were above 30 μg/L, 24/35 (69%) patients had a typical NPC profile, while only 2/12 (17%) patients displaying sertralinemia within lower therapeutic range (between 10 and 30 μg/L) had the same profile (chi‐squared value X^2^ of 20.8, *p* < 0.0001). However, the correlation coefficients between serum sertraline and biomarkers concentrations were relatively low (Spearman's rho = 0.404 for PPCS and 0.403 for C‐triol, *p* = 0.005 for both), suggesting that other factors than sertraline concentrations likely contribute to false‐positive NPC biomarkers profiles. Like in our “pseudo‐NPC” patients, in the sertraline‐treated patients, PPCS was much more elevated than C‐triol (means 6.1 MoC vs. 1.1, as normalized values), and PPCS was more frequently positive than C‐Triol (74% vs. 55% respectively) (Figure 4).

### 3.5 Sertraline Disrupts Intracellular Cholesterol Trafficking in Cultured Fibroblasts

The filipin staining test in fibroblasts allows revealing the accumulation of unesterified cholesterol in subcellular compartments—a hallmark of NPC pathophysiology and has long been the gold standard for NPC diagnosis before implementation of plasmatic biomarkers as first‐line screening tests.

**FIGURE 5:**
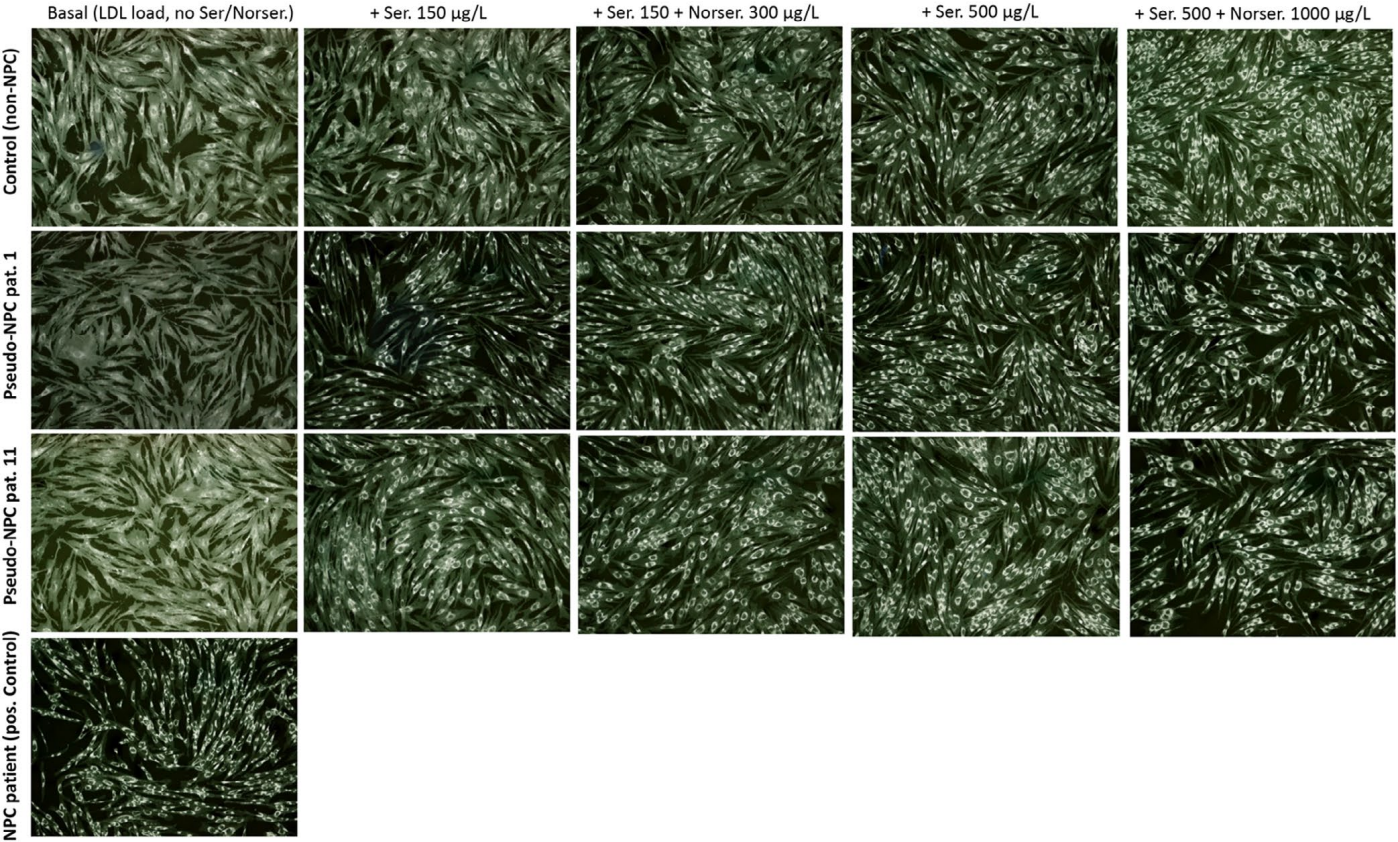
Filipine test in skin fibroblasts after purified human LDL load, without addition of sertraline/norsertraline (basal), and with addition of sertraline +/− norsertraline at therapeutic concentrations (150 and 300 μg/L respectively) and supra‐therapeutic concentrations (500 and 1000 μg/L respectively). Ser: Sertraline Norser: Norsertraline, pat: Patient, pos: Positive.

The test was performed in fibroblasts from 5 pseudo‐NPC patients as part of standard diagnostic care, and was normal (similar to control fibroblasts) in all of them. However, the addition of sertraline to the culture media, with or without its main active metabolite, norsertraline, either at therapeutic or supratherapeutic concentrations, resulted in marked intravesicular fluorescence in the two tested pseudo‐NPC patients, reflecting accumulation of unesterified cholesterol in the endolysosomal compartment (Figure 5). The effect of sertraline was also observed in control fibroblasts (used in the standard filipin staining test as a negative control), and the fluorescence pattern and intensity were very similar to the typical filipin test aspect obtained in fibroblasts from NPC patients (Figure 5).

### 3.6 Biomarker Results in Relation to Other Psychotropic Drugs (“Child Psychiatry” Group)

To gain insight into the potential effects of psychotropic drugs other than sertraline on NPC biomarkers, we studied patients from a Child Psychiatry Department for whom plasma biomarker measurements were available (they were sampled for NPC screening for clinical reasons). Thirty‐three “Child psychiatry” patients were identified, including three pseudo‐NPC patients (patients 3/A, 4/B, and 7/C) (see Table 3). 6/33 were treated with sertraline, the three “pseudo‐NPC” patients and another 3 who had normal NPC biomarkers concentrations. Excluding the three “pseudo‐NPC” patients from the “Child psychiatry” cohort, only 2/30 patients had increased NPC biomarkers concentrations, with very slightly abnormally high C‐triol only. 14 psychotropic drugs were taken by these 30 patients at the timing of biomarkers measurement. Some of these drugs were frequently prescribed (aripiprazole in 12/30, cyamamezine in 10/30); therefore, we can assume these do not lead to increased plasma NPC biomarkers. Apart from sertraline, the only other selective serotonin recapture inhibitor (SSRI) drug taken in the “child psychiatry” cohort was fluoxetine, in two patients that exhibited normal biomarkers profile.

**TABLE 3:** Diagnosis, biomarkers concentrations, and treatments received at the time of blood sampling from the child psychiatry cohort (*n* = 33), including 3 patients also from the pseudo‐NPC cohort, here identified as A (also pseudo‐NPC patient *n*°3), B (also *n*°7) and C (also *n*°4). The biomarkers concentrations were measured between 2023 and 2024.

	Diagnosis	C‐Triol (MoC)	7‐KC (MoC)	PPCS (MoC)	SERT	FLUO	ARIP	CHLO	CLOZ	CYAM	HALD	OLAN	QUET	RISP	LORA	MELA	TROP
1	NK/behavioral disorder	0,3	0,1														
2	Depressive episode	0,4	0,1				×										
3	Mood swings on antidepressants (Sertraline)	0,4	0,5				×			×							
4	Psychotic decompensation	0,8	0,3				×					×			×		
5	Early‐onset schizophrenia	0,7	0,6										×			×	
6	Depressive episode with psychotic features	0,2	0,1	0,2	×					×							
7	Paranoid schizophrenia	0,6	0,9	0,2	×												×
8	Mood disorder	0,6	0,3				×			×							
9	Catatonic syndrome	0,2	0,3				×								×	×	
10	Depressive episode	1,2	0,2														
11	Early onset schizophrenia	0,9	0,5	0,5	×				×								
12	Unknown	0,3	0,2														
A	Obsessive compulsive disorder	4,9	1,6	11,5	×					×				×			
14	Transient acute psychotic disorder	0,3	0,3					×						×		×	×
15	Depressive episode	0,4	0,2				×										
16	Manic episode	0,2	0,1							×							
17	Schizophrenia	0,3	0.5			×	×			×						×	
18	Early onset schizophrenia	0,2	0,1														
B	Obsessive compulsive disorder	1,1	0,7	2,1	×									×		×	
20	Selective mutism	0,6	0,3														
21	Depressive episode	1,1	0,9			×	×	×		×						×	
22	Neurodevelopmental disorder	0,3	0,1				×			×		×				×	
23	Early onset schizophrenia	0,3	< 0,1								×	×			×		
24	Catatonic syndrome	0,6	0,3														
25	Bipolar affective disorder	0,7	0,3				×			×							
26	Depressive episode	0,03	0,2											×		×	
27	Generalized epilepsy with absence	0,3	0,04														
28	Schizophrenia	0,2	0,04														
29	Transient acute psychotic disorder	0,03	0,05							×							
30	Early‐onset schizophrenia	0,5	0,8				×				×			×			
31	Manic episode	0,3	0,2							×		×				×	
32	Oppositional defiant disorder	0,6	0,7				×										
C	Obsessive compulsive disorder	6,3	3,4	1,1	×					×				×		×	

Abbreviations: ARIP, aripiprazole; CHLO, chlorpromazine; CLOZ, clozapine; CYAM, cyamemazine; FLUO, fluoxetine; HALD, haldol; LORA, lorazepam; MELA, melatonin; OLAN, olanzapine; QUET, quetiapine; RISP, risperidone; SERT, sertraline; TROP, tropatepine.

## 4 Discussion

In this study, we reported 15 patients with a similar biochemical profile as observed in NPC patients and no pathogenic variants in *NPC1* or *NPC2*, and found that 13/15 were treated with sertraline. Sertraline (chlorhydrate de (1S,4S)‐4‐(3,4 dichlorophényl)‐1,2,3,4‐tétrahydro‐N‐méthyl‐1‐naphtalamine) is a serotonin (5‐HT) reuptake inhibitor (SSRI), one of the most frequently prescribed antidepressants worldwide, and a first‐line treatment for major depressive disorder, obsessive‐compulsive disorder, and social anxiety disorder among other indications. We subsequently found strong arguments connecting sertraline use and increased NPC biomarkers: (i) in two “Pseudo‐NPC” patients, discontinuing treatment with sertraline was associated with normalization of biomarkers; (ii) sertraline use induced an elevation in plasma concentrations of NPC biomarkers (PPCS, C‐triol and 7‐KC), mimicking the biochemical phenotype of NPC patients in the “sertraline‐treated” patients, with higher levels of sertralin concentration being significantly associated with a greater proportion of patients testing positive for biomarkers; and (iii) sertraline disrupts intracellular cholesterol trafficking in vitro, a hallmark of NPC cellular features, as revealed by filipin test in fibroblasts from non‐NPC individuals (pseudo‐NPC or control).

Pseudo‐NPC patients exhibited a biochemical profile similar to that of genetically confirmed NPC cases; however, their clinical presentation did not match the classic NPC phenotype. The only exception was patient 9, who showed slowed vertical saccades, atypical psychosis, cognitive decline, and ataxia—features highly suggestive of NPC. The filipin staining test, which is typically highly sensitive for diagnosing NPC, was negative. This patient had been receiving long‐term sertraline for nine years. Based on these findings, and considering the functional impact of sertraline on cholesterol metabolism demonstrated in vitro in our study and others (see below), we cannot exclude the possibility that chronic sertraline use may trigger an acquired NPC‐like condition.

About one‐quarter of patients treated with sertraline did not show NPC plasma biomarkers elevation. Interindividual variability in sertraline pharmacokinetics is well documented and is influenced by polymorphisms in cytochrome P450 enzymes, particularly *CYP2C19, CYP3A4*, and *CYP2D6*. Variants in *CYP2C19* and *CYP2B6*, such as the *6 allele associated with reduced enzymatic activity, are present in 10%–15% of individuals of European ancestry and may significantly alter drug metabolism [12, 13]. In addition to pharmacogenetic factors, demographic variables including age, body weight, and gender have also been shown to affect sertraline pharmacokinetics, with shorter elimination times reported in younger males [14, 15]. All these known factors could partially explain the individual susceptibility for sertraline to increase plasma NPC biomarkers, in addition to sertraline dosage. However, additional factors, beyond plasma sertraline concentration, like treatment duration, or the individual impact of sertraline on some metabolic pathways, are probably involved.

Sertraline seems to influence the metabolic pathways of biomarkers involving two lipid classes: oxysterols and N‐acyl‐O‐phosphocholineserine. The link between sertraline and cholesterol metabolism was explored in an in vitro study where cortical neurons and astrocytes were cultured in the presence of sertraline and other antidepressants (aripiprazole, haloperidol, trazodone) [16]. In this study, sertraline administration to neurons and astrocytes led to an increase in 7‐dehydrocholesterol (7‐DHC), a cholesterol precursor. Neurons and astrocytes showed altered sterol composition in response to each medication, with a significant increase in 7‐KC in astrocytes. The elevation of 7‐KC under sertraline, compared to the other molecules tested, was observed at higher concentrations of sertraline (PPCS was not quantified in this study), suggesting that sertraline concentration influences cholesterol metabolism only at a certain threshold. However, in vivo, we did not observe elevated oxysterol levels in patients with child psychiatry diagnoses who were treated with aripiprazole (*n* = 12) or haloperidol (*n* = 2).

There is no clear explanation accounting for the sertraline's effect on plasma NPC biomarkers. We were able to test too few patients taking other SSRIs to comment on this class more generally. But there are relevant published data regarding imipramine, a drug with a similar structure. Imipramine is a cationic amphiphilic drug (CAD). Imipramine and other CADs have been shown to induce an in vitro complex lipidosis via accumulation in late endosomes and lysosomes and to induce a positive filipin test, as we observed here for sertraline [17]. Many drugs are CADs, including drugs used in psychiatry like haloperidol, chlorpromazine, and clozapine, prescribed in different patients from the “child psychiatry” cohort (respectively 2, 2, and 1 patients, 5 in total), without increased NPC biomarkers (except very slightly increased triol in patient 21). There are no available data concerning a potential effect of these three CADs on the filipin test [16]. Altogether, we cannot exclude a CAD class effect of sertraline to explain the effect on NPC biomarkers, but it seems unlikely, and more exhaustive data are needed. Similarly, a general SSRI‐class effect seems unlikely, as no false positive biomarkers were observed in the 2 children treated by fluoxetine (Table 3, cases #17 and #21). Recently, sertraline has been reported to promote cholesterol accumulation within lysosomes, resulting in lysosomal membrane permeabilization, disruption of autophagy, and cell death [18]. The mechanism suggested by the authors was the inhibition of cholesterol binding to the lysosomal cholesterol transporters NPC1 and NPC2, as indicated by molecular docking analysis. This mechanism could perfectly explain our findings, but experimental data are needed.

Interestingly, there is a recent report of [19] nine cases of late‐onset multiple acyl‐CoA dehydrogenase deficiency (MADD) who lacked the typical pathogenic variants associated with that disorder; 7 of these patients were being treated with sertraline and improved clinically following treatment discontinuation. This study also suggested a dose‐dependent effect (> 100 mg) with several patients presenting symptoms shortly after dosage increase.

Patients 10 and 13 from our “pseudo‐NPC” cohort had never received sertraline treatment. Patient 10 was also the only one presenting with severe liver disease with cholestasis and elevated cholestanol levels, without psychiatric or neurological symptoms. A correlation between elevated plasma oxysterols and liver involvement in NPC patients has been demonstrated, particularly by the relationship between increased bilirubin and oxysterol levels [11, 20]. In the case of our patient, oxysterol elevation may therefore be explained by cholestasis and liver involvement. We have no data on the relationship between cholestasis and PPCS concentrations in the literature to date. PPCS has been observed to be elevated in glycosylation disorders, particularly in a patient with *ATP6AP1* deficiency associated with cholestasis and liver disease, although no correlation between liver enzymes and PPCS concentrations could be established [21].

According to ClinCalc DrugStats (derived from MEPS data), Sertraline ranked 11th among medications prescribed in the U.S, in 2022 and received an estimated 42.6 million prescriptions in 2023 [22]. In this context, our study is significant for interpreting NPC biomarkers concentrations in the diagnostic approach to NPC disease. Indeed, we demonstrated that sertraline can induce increased concentrations of the three NPC biomarkers significantly above the upper normal limit in more than 50% of cases, which can lead to costly and unnecessary investigations or even erroneous diagnosis of NPC disease. This may have significant implications for the prognosis and management of patients and could lead to the unnecessary administration of complex treatments. It is therefore essential to consider sertraline influence when interpreting plasma NPC biomarkers, and always perform *NPC1* and *NPC2* gene analysis to establish the diagnosis of NPC. Moreover, if sertraline does inhibit binding of cholesterol to NPC1 and NPC2 proteins as it is suspected, clinical long‐term effect of such mechanism of action mimicking NPC pathophysiology should be carefully monitored.

## Author Contributions

Maria Makrygianni contributed to the conception and design of the study, data acquisition and analysis, and drafting significant portions of the manuscript. Cécile Pagan contributed to study design, data acquisition and analysis, and drafting significant portions of the manuscript. Antony Citterio‐Quentin contributed to study design, data acquisition, and critical review of the manuscript. Isabelle Rouvet, Giulia Dingeo, Cyril Hanin, Claudine Laurent‐Levinson, Samia Pichard, Bénédicte Héron, Clément Gourguechon, Daniele Mandia, Jérôme Guitton, Carole Lacout, Nicolas Schleinitz, Florient Potier, Olivier Flabeau, and Elsa Besse‐Pinot contributed to data acquisition and critical review of the manuscript. Florent Lamari contributed to study design, data acquisition, and critical manuscript revision. Magali Pettazzoni contributed to study design, data acquisition and analysis, and drafting significant portions of the manuscript. Yann Nadjar contributed to the conception and design of the study, data acquisition and analysis, and drafting significant portions of the manuscript.

## Funding

The authors have nothing to report.

## Conflicts of Interest

The authors declare no conflicts of interest.

## Data Availability

The data that support the findings of this study are available on request from the corresponding author. The data are not publicly available due to privacy or ethical restrictions.
